# Characteristics of Patients With Trigeminal Neuralgia Referred to the Indonesian National Brain Center Neurosurgery Clinic

**DOI:** 10.3389/fsurg.2021.747463

**Published:** 2022-01-10

**Authors:** Mustaqim Prasetya, Peter Adidharma, Adi Sulistyanto, Takuro Inoue, Abrar Arham

**Affiliations:** ^1^Department of Neurosurgery, National Brain Center Hospital, East Jakarta, Indonesia; ^2^Department of Neurosurgery, Subarukai Koto Memorial Hospital, Higashiomi, Japan

**Keywords:** trigeminal neuralgia, facial pain, Indonesia, neurosurgery clinic, epidemiology

## Abstract

Trigeminal neuralgia (TN) is a debilitating neuropathic pain involving the fifth cranial nerve. There has been no study investigating the clinical and socioeconomical characteristics of patients with TN in Indonesia. A total of 100 patients were included in this study. Symptoms indicating a later stage of the illness, namely, involvement of all the trigeminal nerve branches, numbness, and concomitant persistent pain, were the common presentations found in our cohort. Only one TN diagnosis was made by a general practitioner (GP). None were immediately referred to a neurosurgeon following their diagnosis. Access to our clinic took as long as 4.7 ± 5.1 years (mean ± SD) from the onset. Older age was a significant predictor of an increased likelihood of not knowing their illness upon the referral (21.9%, *p* = 0.008). Upon their first presentation, 25.5% of patients had experienced drug-related side effects due to prolonged medication. Only 50% of patients were compensated by the universal health coverage (UHC) system. Seven patients spent ≥ 50 million rupiahs and eight patients had already lost their jobs. In conclusion, early contact with a neurosurgeon contributes to better management of TN, both for the patients and healthcare system in Indonesia. A refined understanding of TN nature is still needed in this country.

## Introduction

Trigeminal neuralgia (TN) is a neurologic disease characterized by a sudden and triggered-bout severe pain in the sensory area of the fifth cranial nerve (1). Due to the severity of the pain, debilitating effects of TN on the life of patient, either physically, mentally, or socioeconomically, have been numerously documented (2–6).

Since first described in 1756, treatment methods of TN have been continuously developing. One of the most significant changes was the establishment of surgical treatment of TN. The procedure, called microvascular decompression (MVD), was popularized by Peter Jannetta and has been the only definitive treatment of TN to date. The pain resolution after MVD is expected in more than 76% of patients, even at 10 years following the surgery (7, 8). Furthermore, improved surgical instruments and techniques had achieved even better pain control in the recent studies (9–11). This advancement has emphasized the integral role of a neurosurgeon in the management of TN.

In 2014, Indonesia, a developing middle-income country, introduced its novel universal health coverage (UHC) system, covering more than 203 million people (12). Also, within the same year, first multidisciplinary, tertiary-level neurological center in Indonesia, the Indonesian National Brain Center, started to operate, commencing to provide a wide variety of neurology-related healthcare to the public. These establishments have given access to the curable opportunity with neurosurgical procedures for patients with TN.

An established epidemiological study is a vital foundation for clinical and public health practices. This basis is the gap of knowledge in achieving the utmost management of TN in Indonesia. Through epidemiological study, diagnostic accuracy can be improved and risk factors, disease progression pattern, and burden among the affected population can also be identified (13, 14). This study aims to develop the landscape of patients with TN in Indonesia who were referred to the Indonesian National Brain Center. Sharing this information among the medical professionals involved in treating TN may allow healthcare systems of other developing countries to manage patients with TN efficiently.

## Methods

### Study Subjects

Consenting patients diagnosed with TN by the International Classification of Headache Disorders-3rd Edition (ICHD-3) criteria were enrolled in this study between April 2014 and January 2021. Consecutive sampling was employed. All of them were attending the Indonesian National Brain Center neurosurgery clinic in Jakarta, Indonesia.

### Ethics Approval

This study has been done with prior written consent after an information sheet has been provided to all the subjects. Approval by the Indonesian National Brain Center Hospital Ethics Committee was obtained.

### Data Collection

Data were collected through a checklist-guided interview by an attending neurosurgeon (MP), which consisted of the socioeconomic, demographical, and clinical characteristics of patient. Prior medical consultation and treatment concerning the facial pain were also noted including who made the diagnosis, frequency and economic burden of prior treatment, its effect, and knowledge of patient with respect to their illness. The pain history was also collected: onset, duration, side of pain, trigger, frequency, pain quality, severity, and progression. Effect of TN on the social-economical domain of patient was also documented. Data from electronic medical record (EMR) of patient was also included as an addition.

### Statistical Analysis

Statistical analyses were conducted by independent physicians in R statistical environment (version 1.3.1093). The socioeconomic and clinical characteristics of patient were descriptively presented. The mean was expressed with ±SD, while the median was expressed with interquartile range (IQR) (first quartile to third quartile). The Shapiro–Wilk test was employed for normality testing. The chi-squared, Fisher's exact, Mann–Whitney *U*, Spearman's, Pearson's, and linear regression tests were used accordingly. A *p* < 0.05 was considered to indicate a statistically significant association or correlation.

## Results

### Demographical Characteristics

A total of 100 subjects were included, with some variables were missing for some of patients. The demographical data are given in Table 1. The mean age (SD) at diagnosis was 55.2 (13.8) years, with female predominance (59%). Of these, 22% of patients (*n* = 22/100) either had less than a high-school diploma or illiterate and 90% of patients (*n* = 90/100) lived within the island. A total of 90 patients (*n* = 90/100, 90%) of the cohort were head of the family and 24% of patients (*n* = 24/100) were full-time workers. Hypertension was found in 42 patients (*n* = 42/100, 42%), where more than half patients (*n* = 26/42, 62%) were females. No multiple sclerosis was identified and 10 patients (*n* = 10/100, 10%) were smokers.

**Table 1:** Basic demographical data.

**Characteristic**	**Value**
Age at diagnosis—year^*^	55.2 ± 13.8
Female sex—*n* (%)	59 (59%)
Ethnicity—*n* (%)	
Javanese	39 (39)
Betawi	17 (17)
Sundanese	17 (17)
Bataknese	7 (7)
Others^†^	20 (20)
Educational level—*n* (%)	
Bachelor's/master's degree	13 (13)
Associate degree	10 (10)
High school graduate	55 (55)
Less than a high school diploma	19 (19)
Illiterate	3 (3)
Marital status—*n* (%)	
Married	85 (85)
Separated	6 (6)
Single	5 (5)
Divorced/widowed	4 (4)
Head of family—*n* (%)	90 (90%)
Working status—*n* (%)	
Homemaker	34 (34)
Full-time worker	24 (24)
Not working	17 (17)
Retired	13 (13)
Part-time worker	9 (9)
Student	3 (3)
Living within the Java Island—*n* (%)	90 (90%)
Previous medical history—*n* (%)	
Hypertension	42 (42)
Ischemic stroke	9 (9)
Migraine headache	5 (5)
Diabetes Mellitus type 2	3 (3)
Others^‡^	76 (76)
Smoking history—*n* (%)	10 (10)

* *Mean ± SD*.

† *Others, Bengkulunese; Buginese; Chinese; Manadonese; Medanese; Melayunese; Minangnese; Palembangnese*.

‡ *Others, dyspepsia syndrome; chronic kidney disease; facial trauma; malignancy; hyperuricemia; benign paroxysmal positional vertigo*.

### Pain Characteristics, Severity, Progression, and Remission

The right side was most commonly affected (*n* = 51/97, 52.6%), with involvement of all (V1, V2, and V3) the trigeminal branches being the most common to be seen in our cohort (*n* = 26/96, 26.5%). The patients with episodes of paroxysmal pain mostly experience 2–10 episodes of daily pain (*n* = 46/73, 63%), with a duration of 2–30 mins seen in 57.1% of patients (*n* = 52/91). A total of 77 patients (*n* = 77/91, 84.6%) complained of having paroxysmal pain lasting more than 2 min, which was significantly associated with a longer duration of illness (Mann–Whitney *U* test, *p* = 0.023, median = 2.5 vs. 4.5 years).

Numbness in any division of trigeminal nerve was observed in 28 patients (*n* = 28/96, 29%), even though only two of them had undergone invasive procedures (one patient underwent MVD and one patient underwent percutaneous radiofrequency rhizotomy). However, 30% of patients with TN with numbness had a previous history of ischemic stroke, which was statistically significant (Fisher's exact test, *p* = 0.002) vs. 3% in patients with TN without numbness. The mean duration from the onset was longer among patients with numbness (5.3 vs. 4.6 years), although it was not statistically significant. Table 2 presents the other clinical characteristics.

**Table 2:** Characteristic of facial pain.

**Characteristic**	**Value**
Side of pain—*n* (%)	
Right	51 (52.6)
Left	44 (45.4)
Bilateral	2 (2.1)
Trigeminal nerve branch involved—*n* (%)	
V1^*^	5 (5.1)
V2^*^	19 (19.4)
V3*	22 (22.4)
V1–V2^*^	10 (10.2)
V2–V3^*^	14 (14.3)
V1–V2–V3^*^	26 (26.5)
Types of facial pain—*n* (%)	
Purely paroxysmal	85 (85)
Paroxysmal with concomitant continuous pain	8 (8)
Purely concomitant continuous pain	7 (7)
Daily attack frequency—*n* (%)	
Single episode	5 (6.8)
2–5 episodes	23 (31.5)
6–10 episodes	23 (31.5)
>10 episodes	15 (20.5)
Variable frequency	7 (9.6)
Duration of each pain episode—*n* (%)	
<2 mins	14 (15.4)
2–30 mins	52 (57.1)
30–60 mins	9 (9.9)
>60 mins	14 (15.4)
Variable duration	2 (2.2)
Quality of pain—*n* (%)	
Electric shock-like pain	65 (65)
Sharp shooting pain	45 (45)
Stabbing pain	35 (35)
Burning pain	32 (32)
Throbbing pain	28 (28)
Others^†^	5 (5)
Pain attack triggered by—*n* (%)	
Chewing	85 (85)
Talking	80 (80)
Brushing teeth	78 (78)
Washing face	74 (74)
Blowing wind	60 (60)
Cold temperature	53 (53)
Shaving^‡^	40 (97.6)
Numbness in any division of trigeminal nerve—*n* (%)	28 (29)

* *V1, ophthalmic branch; V2, maxillary branch; V3, mandibular branch*.

† *Others, tingling sensation; numb*.

‡ *Only counted in males*.

Table 3 depicts pain severity and its progression. Upon contact at the neurosurgery clinic, the median (1st quartile to 3rd quartile) numeric rating scale (NRS) for pain severity was 8 (6–9) and 71.7% of patients (*n* = 71/99) were having severe pain according to verbal rating scale (VRS) classification. A total of 40 patients (*n* = 40/97, 41.2%) had either severe pain without any relief with medication or not adequately controlled by medication [Barrow Neurological Institute (BNI) pain scale IV or higher]. Pain progression in terms of frequency and severity was seen in 76.5 and 74.7% of patients, respectively. An increase in severity and frequency was significantly associated with one another (Fisher's exact test, *p* < 0.0001). A progression in pain frequency was significantly associated with younger age (≤ 51-year-old, chi-squared test, *p* = 0.02). However, younger age was not significantly associated with a progression in the pain intensity and no other clinical or socioeconomical factors were significantly associated with pain progression. In 40.4% (*n* = 40/99) of patients, a period of complete remission for at least 2 weeks was seen.

**Table 3:** Pain severity, progression, and remission.

**Characteristic**	**Value**
Numeric rating scale (NRS)^*^	8 (6–9)
Verbal rating scale (VRS)—*n* (%)	
Mild	12 (12.1)
Moderate	16 (16.2)
Severe	71 (71.7)
Barrow Neurological Institute pain scale—*n* (%)	
I—No pain, no medication required	0 (0)
II—Occasional pain, no medication required	7 (9.3)
III—Some pain, adequately controlled by medication	50 (51.5)
IV—Some pain, not adequately controlled by medication	33 (34)
V—Severe pain or no pain relief with medication	7 (7.2)
Increasing pain frequency—*n* (%)	75 (76.5)
Increasing pain severity—*n* (%)	74 (74.7)
Persistent pain location—*n* (%)	76 (80)
Period of remission^†^–*n* (%)	40 (40.4)

* *Median (1st quartile to 3rd quartile)*.

† *Period of complete remission for at least 2 weeks in duration*.

### Previous History of Pain-Related Consultations

In total, 99% (*n* = 99/100) of the diagnoses were made by specialists at a secondary or tertiary healthcare facility, with most of them (*n* = 85/100, 85%) were made by a neurologist. Even though 56% (*n* = 56/100) of all the patients had a previous history of consultation with a general practitioner (GP) at a primary healthcare facility, only one patient was diagnosed by them. Before referral, 31% of patients (*n* = 31/100) had more than four consultations with a healthcare professional. Even though, 43 patients (*n* = 43/100, 43%) of the cohort had a previous visit with a neurosurgeon and none of them were immediately referred to a neurosurgeon upon diagnosis (Table 4). The mean duration (SD) from the onset to referral to our clinic was 4.7 (5.1) years. Ten of them (*n* = 10/99, 10.1%) were referred 10 years or longer after the pain onset. In total, 37 of our patients (*n* = 37/100, 37%) who had ≥ 20 years of life expectancy (≤ 51 years) had a mean duration of 5.6 years from onset to first contact with our clinic, which is 0.9 years longer than the entire cohort, although it is not statistically significant.

**Table 4:** Previous history of medical consultation.

**Characteristic**	**Value**
Previous history of consultation with—*n* (%)	
Neurology specialist	71 (71)
Dentist and oral-maxillofacial surgeon	69 (69)
General practitioner	56 (56)
Neurosurgeon	43 (43)
Acupuncture specialist	13 (13)
Otorhinolaryngology specialist	12 (12)
Herbal medicine	9 (9)
Others^*^	5 (5)
Number of previous consultations—*n* (%)	
≤ 5	56 (64.4)
6–10	8 (9.2)
11–20	8 (9.2)
21–30	5 (5.7)
>30	10 (11.5)
Diagnosed as trigeminal neuralgia by—*n* (%)	
Neurology specialist	85 (85)
Neurosurgeon	8 (8)
Dentist and oral-maxillofacial surgeon	4 (4)
General practitioner	1 (1)
Orthopedic surgeon	1 (1)
Duration from onset to referral^†^–year	4.7 ± 5.1

* *Others, ophthalmology specialist; dermatovenereology specialist; internal medicine specialist; orthopedic surgeon*.

† *Mean ± SD*.

At the first contact in our neurosurgery clinic, as much as 21.9% (*n* = 21/96) of the cohort did not know their diagnosis. The logistic regression analysis was performed to ascertain the effects of demography, clinical characteristics, and previous consultation factors. The model was statistically significant (χ^2^ = 20.754, *p* = 0.008, Nagelkerke *R*^2^ = 32.7%) and increasing age was significantly associated (*p* = 0.018) with an increased likelihood of not knowing their diagnosis.

### Previous History of Pain-Related Treatment and Its Outcome

Carbamazepine was the most prescribed drug among the patients initially treated with medication (*n* = 95/100, 95%). Five patients (*n* = 5/100, 5%) had no previous history of medication for pain relief. More than a quarter patients (26.3%, *n* = 25/95) had already experienced drug-related side effects upon referral. In total, 24 patients (*n* = 24/100, 24%) had a previous history of invasive procedures, with percutaneous radiofrequency rhizotomy being the most frequent (33.3%, *n* = 8/24). Previously prescribed drugs and invasive procedures performed for pain relief are given in Table 5. Only six patients (*n* = 6/99, 6.1%) reported complete relief of pain from previous treatment. Most of them only had partial pain relief (*n* = 58/99, 58.6%), while 22 patients (*n* = 22/99, 22.2%) had no resolution and 13 patients (*n* = 13/99, 13.1%) experienced worsening of pain.

**Table 5:** Previous treatment history.

**Characteristic**	**Value**
Previously consumed drug—*n* (%)	
Carbamazepine	75 (75)
Gabapentin	25 (25)
NSAID	5 (5)
Paracetamol	4 (4)
Opiate analgesic	3 (3)
Amitriptyline	3 (3)
Others^*^	8 (8)
No previous drug consumption	5 (5)
Experiencing drug related side effect—*n* (%)	25 (25.5)
Previous invasive procedure for pain relief—*n* (%)	
Percutaneous RF rhizotomy^†^	8 (8)
Tooth extraction	6 (6)
Peripheral block	5 (5)
Acupuncture	3 (3)
MVD^‡^	2 (2)
No previous invasive procedure	76 (76)

* *Others, oxcarbamazepine; methylcobalamin; phenytoin; oral corticosteroid; lamotrigine; valproic acid*.

† *RF, radiofrequency*.

‡ *Microvascular decompression*.

### Social and Economic Impact of TN in Indonesia

A total of 13 patients (*n* = 13/91, 14.4%) reported significant disturbance in work productivity, with 11 patients (*n* = 11/91, 12.2%) losing jobs due to the facial pain. In total, 52 patients (*n* = 52/91, 57%) reported no effect on working productivity, while 26 patients (*n* = 26/91, 28.6%) complained of decreased work productivity. Effect of pain on work was significantly associated with the previous treatment outcome (chi-squared test, *p* = 0.0155). Five patients (*n* = 5/11, 45.5%) who lost their jobs reported pain worsening as previous treatment outcomes.

The coverage ratio by the Indonesia UHC scheme was only 50% (*n* = 48/96) in our cohort. Among the UHC-uncovered patients, 31 patients (*n* = 31/48, 64.6%) had spent more than 5 million rupiahs (in Indonesian Rupiah—IDR, 1 US dollar equals to 14,495 IDR) and 7 patients (*n* = 7/48, 14.6%) spent more than 50 million rupiahs (3,490.66 USD) for their treatment. There was a moderate, positive correlation between health expenditure and duration from onset to referral, which was statistically significant (Pearson's test, *r* = 0.4927, *n* = 46, *p* = 0.0005).

## Discussion

This study revealed identical clinical characteristics of patients with TN with those outsides of Indonesia (1, 15, 16), except for some differences that will be discussed below.

A low prevalence of bilateral involvement was noted in this study (2.1%). This difference may be related to the low prevalence of multiple sclerosis (MS) in Indonesia and other Asian populations compared to the Caucasian population (17). Bilateral TN has been implicated previously in up to 30% of patients with MS in the literature (18–20). The low prevalence of MS in Indonesia may be related to the low number of bilateral patients with TN in our cohort.

Involvement of all the trigeminal nerve branches was distinctly the most common presentation. It is contradictory to many previous findings, which stated that only the second and third trigeminal nerve branches were affected in most cases (1, 15, 16, 21, 22). It is possibly caused by the late referral to our center, allowing the disease to progress. Extension to the first division of the trigeminal nerve has been reported as a manifestation of progression of TN (15, 16, 21).

Moreover, we encountered a surprisingly high proportion (29%) of patients who complained of numbness in the sensory area of the trigeminal nerve. It was presumably another sign of progression of TN observed in our cohort. Numbness has been previously depicted as a sign of deteriorated TN in several studies (23–26). With only two patients having a previous history of either percutaneous rhizotomy or MVD, the association between invasive procedures and numbness that has been previously reported was not seen in this study (24). Numbness, however, was linked to late referrals that led to disease progression.

Furthermore, paroxysmal pain lasting more than 2 mins was documented in 35.7% of patients. Haviv et al. postulated that more prolonged paroxysmal pain is related to the duration of the illness (27), a pattern we also similarly had in our cohort. In our opinion, patients with TN should be referred to a neurosurgeon at the early stage, before the illness progresses, as they can be benefited from a variety of therapeutic choices, including MVD.

Poor recognition of TN among patients and physicians is an issue that we faced in supporting an ideal healthcare system. Poor recognition of their illness from the side of patient is a barrier for medical practitioners in providing the necessary medical services at proper timing. Almost a quarter of our patients did not recognize their illness at the first contact in our neurosurgery clinic. Older age was identified as an independent risk factor for lack of recognition of their disease in this study. Previous studies have shown that literacy of a person on their disease is crucially determining how they cope with pain and affecting their treatment outcome (28–31). Our observation encourages the medical practitioners to inform a comprehensive knowledge of patient about TN. On the other hand, only one patient was diagnosed by a GP, amidst more than half of the cohort had previous consultations with one. Despite the difficulty in recognizing TN among GPs has been previously reported (32–34), TN can be clinically diagnosed with careful history taking even considering its variability in presentation. Thus, our epidemiological study revealed a poor understanding of the illness on both the patients and the medical professionals. Early contact between patients and neurosurgeons is essential in achieving excellent care for potentially debilitating TN and for preventing further socioeconomical loss at individual and public health levels. We believe that it is crucial to make a prompt diagnosis at first contact of patient with GPs in the primary healthcare facility and enhance community knowledge with respect to TN to improve its treatment in Indonesia.

The financial aspect of managing TN is remarkably worrisome in our country. In our cohort, the UHC covered only 50% of patients. This finding was contradictory with the national data of coverage of the system, which has been reported to reach 82.51%. We found no explanation for this matter, as all the expenditures were supposed to be covered, including dental and oral-maxillofacial surgery treatment (35). Since 14.6% of patients not covered by the UHC spent more than 50 million rupiahs in a country with a GDP per capita of 56.9 million rupiahs (in 2020), further study is needed to dwell deep on this matter (36). The Indonesian National Brain Center has provided excellent neurosurgical services covered by the UHC since its establishment in 2014. However, this study revealed that most patients were referred at the later stage of the illness. We were not able to estimate the social loss from prolonged ineffective medications for medically intractable patients with TN. However, numerous patients in our cohort suffered socioeconomically from decreased productivity at work and even lost their jobs besides suffering from the pain itself.

All of the issues we encountered here, namely, advanced TN at initial presentation, underdiagnosis by GPs at primary healthcare facilities, poor disease knowledge among patients, and high socioeconomic burden, points to ineffective management of TN at a public health level. The shortage of neurosurgeons and an unequal geographical distribution are issues that limit neurosurgical access for patients with TN. According to the Indonesian College of Neurosurgeons, our country has a ratio of one neurosurgeon for every 687.5 thousand people, compared to one for every 60–80 thousand people in developed nations (37). More than 15% are concentrated within capital city of Indonesia and almost one-third provinces of Indonesia do not have access to neurosurgical services. Early contact with a neurosurgeon in rural areas is almost impossible and geographical barriers between islands make referrals to our center difficult and expensive. Thus, it is not surprising that only 10% of patients with TN were from outside the island.

The UHC stakeholders must provide at least one comprehensive consultation with a neurosurgeon upon diagnosis. A consensus when to perform an MVD must also be reached. According to a study by Linskey et al., we advocate that all the patients with TN should immediately be referred to a neurosurgeon upon diagnosis. This would allow for judicious surgical decision-making including considerations on risks and appropriate timing (38). We believe that this is feasible under the current UHC system in Indonesia.

Complex MVD surgery, such as in cases such as vertebrobasilar dolichoectasia, must be conducted centrally at a national referral center such as ours and a clear referral pathway must be constructed by the UHC stakeholders. The current system seems to underutilize our resources to perform complex MVD surgeries. Regionalization for simple MVD cases is ideal, but an unequal distribution of neurosurgeons, surgical instruments, and MRI facility prevents the realization of such schemes (39). We believe that implementation of such systems would improve national outcomes of TN, both clinically and socioeconomically.

In this study, we present a clinical and socioeconomical landscape of patients with TN in a developing country, with unusual features of disease progression and a high socioeconomic burden. The challenges and lessons learned at a public health level can be applied to other developing countries, allowing them to design effective healthcare schemes for comprehensive management of TN. However, differences in geographical barriers and healthcare systems must be considered while interpreting our results and opinions.

## Conclusion

In conclusion, we presented the characterization of TN in Indonesia including the impact and challenges we faced at social, economic, and healthcare system aspects. However, it was only conducted in a tertiary healthcare setting, making our findings may not be representable to all the populations with TN in Indonesia. Our sample size was also limited. A further study was conducted at every healthcare facility level, with larger sample size, using validated instruments, assessing deeply on its health economy domain, including follow-up until the postoperative period is still needed. Current system in Indonesia has enabled an ideal scheme for management of TN to be applied, yet its realization remains a challenge to be addressed.

## Data Availability Statement

The raw data supporting the conclusions of this article will be made available by the authors, without undue reservation.

## Ethics Statement

The studies involving human participants were reviewed and approved by Indonesia National Brain Center Ethical Committee. The patients/participants provided their written informed consent to participate in this study.

## Author Contributions

MP, AS, and PA contributed to the conception, design, and acquisition of the study. MP, PA, and TI conducted the data analysis and wrote the manuscript. All authors contributed to manuscript revision, read, and approved the submitted version of the manuscript.

## Conflict of Interest

The authors declare that the research was conducted in the absence of any commercial or financial relationships that could be construed as a potential conflict of interest.

## Publisher's Note

All claims expressed in this article are solely those of the authors and do not necessarily represent those of their affiliated organizations, or those of the publisher, the editors and the reviewers. Any product that may be evaluated in this article, or claim that may be made by its manufacturer, is not guaranteed or endorsed by the publisher.
